# DNA Extraction Optimisation for Minute Land Snails of *Vertigo* Müller, 1773 (Gastropoda: Vertiginidae): A Comparative Evaluation of Six Methods, Including a Non‐Destructive Shell‐Preserving Protocol

**DOI:** 10.1002/ece3.74295

**Published:** 2026-09-04

**Authors:** Benediktas Jukonis, Jekaterina Havelka, Grita Skujienė

**Affiliations:** ^1^ Department of Zoology, Institute of Biosciences, Life Science Center Vilnius University Vilnius Lithuania

**Keywords:** conservation genetics, Habitats Directive, HotSHOT, micromolluscs, non‐destructive sampling, voucher specimen

## Abstract

No systematic comparison of DNA extraction strategies exists for minute Vertiginidae (shell height < 3 mm), a group posing a dual analytical challenge: extremely low tissue input and co‐purified PCR‐inhibitory mucus. For legally protected species, an additional requirement to preserve the shell voucher further constrains available protocols. Using *Vertigo antivertigo* as the model species, we compared six approaches applied to specimens preserved in 96% ethanol (*n* = 10 per method): two HotSHOT alkaline‐lysis protocols (destructive and non‐destructive shell‐preserving variants), a modified CTAB protocol supplemented with PVP‐40 and DTT, and three commercial silica‐column kits (GeneJET Genomic, DNeasy Blood & Tissue, QIAamp DNA Micro). DNA yields were quantified by QuantiFluor fluorometry, and PCR performance was subsequently assessed across four loci (COI barcode, COI mini‐barcode, ITS1, ITS2). DNeasy Blood & Tissue produced the highest fluorometric concentrations; QIAamp DNA Micro and CTAB + PVP‐40 gave intermediate values. The shell‐preserving HotSHOT variant yielded lower concentrations but improved A260/230 ratios. BSA and trehalose supplementation increased PCR success in inhibition‐prone HotSHOT extracts from 70% to 100%. ITS1 Sanger sequencing of three *Vertigo* species listed in Annex II of the EU Habitats Directive, all extracted with the shell‐preserving protocol, confirmed species‐level identification (99.8%–100% BLASTn identity; mean Phred *Q* > 51). The shell‐preserving non‐destructive HotSHOT protocol yields sequenceable DNA from protected Vertiginidae while retaining the morphological voucher, making it the preferred option for conservation‐genetic monitoring. The practical decision framework documented here—integrating voucher preservation, amplification robustness and per‐sample cost—has broad applicability to other minute terrestrial gastropods processed in large‐scale biodiversity surveys.

## Graphical abstract

We compared six DNA extraction methods for minute Vertigo land snails, including a rapid non‐destructive HotSHOT protocol that preserves shells. The shell‐preserving protocol produced sequenceable DNA from very small and conservation‐relevant specimens while retaining voucher morphology. The workflow supports molecular studies of Vertigo where destructive sampling is undesirable.

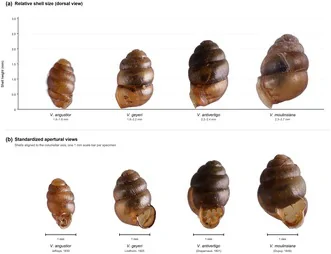

## 1 Introduction

Molecular methods have become integral to terrestrial gastropod systematics, phylogeography and conservation biology since the advent of DNA barcoding (Hebert et al. 2003; Davison et al. 2009) and remain widely used in modern land snail biodiversity and conservation studies (Mohammadi and Ahmadzadeh 2024). Their utility is pronounced in Vertiginidae Fitzinger, 1833, minute Holarctic land snails (Cameron and Pokryszko 2005), where several species are morphologically cryptic and reliable determination from shell characters alone is frequently unattainable, particularly among juveniles (Nekola et al. 2018; Horsák et al. 2024). Four *Vertigo* species are listed in Annex II of the EU Habitats Directive (Council Directive 92/43/EEC): *Vertigo angustior* Jeffreys, 1830; *Vertigo genesii* (Gredler, 1856); *Vertigo geyeri* Lindholm, 1925; and *Vertigo moulinsiana* (Dupuy, 1849). This listing obliges Member States to designate Special Areas of Conservation and to conduct periodic monitoring of conservation status (Horsák 2006; Coufal et al. 2024), generating a recurring demand for reliable and scalable molecular workflows.

The practical consequences of these constraints are starkly illustrated by the most comprehensive molecular study of the genus to date: Nekola et al. (2018) sequenced 424 *Vertigo* individuals across 91 taxa using four gene regions (ITS1, ITS2, CytB, 16S), but because proteinase K diffuses poorly into the tiny, tightly coiled shells, every specimen had to be destroyed for DNA extraction using the Omega BioTek Mollusc DNA Extraction Kit. That study's reliance on complete shell destruction underscores the tension between molecular and morphological data acquisition in minute gastropods and motivates the search for non‐destructive alternatives.

Two interrelated constraints set DNA work with minute Vertiginidae apart from comparable efforts in larger gastropods. Body wet mass seldom exceeds 1 mg, precluding selective dissection of foot tissue; the entire soft body must therefore serve as extraction input, elevating the contaminant‐to‐DNA ratio. Gastropod mucus is dominated by high‐molecular‐weight mucins and glycosaminoglycans, which give it a markedly viscous character that impedes homogenisation and pipetting of low‐volume lysates; together with acidic polysaccharides and polyphenolic proteins, these macromolecules co‐purify with nucleic acids and inhibit downstream enzymatic reactions, a long‐recognised obstacle in molluscan molecular biology (Winnepenninckx et al. 1993; Skujienė and Soroka 2003; Adema 2021; Efstratiou et al. 2025). The severity of this problem scales inversely with body size: whereas larger gastropods permit excision and rinsing of a defined tissue block, which lowers the ratio of mucus to tissue entering the lysis buffer, in Vertiginidae the entire body including the mantle and mucus glands must be used.

Preservation history imposes a second and independent constraint. In ethanol‐preserved museum material, PCR success in molluscs is strongly governed by specimen age and amplicon length, with short fragments typically outperforming standard COI barcodes (Jaksch et al. 2016; Goulding 2021). Ancient DNA recovered from empty shells has extended the temporal reach of molluscan molecular studies to museum specimens spanning decades to millennia (Villanea et al. 2016; Der Sarkissian et al. 2020; Psonis et al. 2022), but these approaches typically target degraded templates and require specialised clean‐room facilities, placing them outside the scope of routine conservation genetics.

Selecting an extraction strategy entails balancing cost, throughput, inhibitor removal and specimen conservation. The foundational CTAB protocol developed by Winnepenninckx et al. (1993) established hexadecyltrimethylammonium bromide as the reagent of choice for molluscan DNA because it forms insoluble complexes with polysaccharides that partition into the organic phase during chloroform extraction. Subsequent modifications—notably the addition of PVP‐40 to sequester polyphenolic compounds (Porebski et al. 1997; Chakraborty et al. 2020)—have improved purity further, and Chakraborty et al. (2020) demonstrated that modified CTAB outperformed commercial kits in terms of reproducibility and sequence quality for non‐marine molluscs. At the other end of the complexity spectrum, the HotSHOT alkaline lysis protocol (Truett et al. 2000) provides low per‐sample cost and rapid processing but may yield inhibitor‐laden, fragmented DNA. Commercial silica‐column kits occupy an intermediate position, trading higher reagent costs for standardised workflows and reduced hands‐on time (Pereira et al. 2011; Schenk et al. 2023). For scarce or legally protected specimens, destructive extraction eliminates the morphological voucher; a non‐destructive approach yielding PCR‐quality DNA would permit simultaneous acquisition of molecular and morphological data from the same individual (Armbruster et al. 2005; Zieritz et al. 2018; Ferreira et al. 2020; Martin et al. 2021). Non‐lethal alternatives such as FTA‐card sampling of foot mucus have shown promise for larger gastropods (Leung et al. 2024) but are impractical for Vertiginidae, whose minute body size precludes reliable mucus collection without handling damage.

Despite this need, no systematic head‐to‐head evaluation of extraction methods—including a shell‐preserving option—has been published for Vertiginidae. The existing method‐comparison literature for molluscs has focused predominantly on larger taxa (Pereira et al. 2011; Chakraborty et al. 2020), museum wet‐preserved collections (Jaksch et al. 2016), or ancient/subfossil material (Villanea et al. 2016; Psonis et al. 2022), leaving a methodological gap for minute, ethanol‐preserved gastropods that form the bulk of conservation‐genetic screening. Our objectives were therefore to: (1) compare six extraction approaches, including a shell‐preserving non‐destructive HotSHOT variant, applied to *V. antivertigo* preserved in 96% ethanol, quantifying DNA concentration and spectrophotometric purity for each; (2) evaluate PCR amplification success across four loci (COI, COI mini‐barcode, ITS1, ITS2) under standard PCR conditions; (3) characterise the fragment‐size distribution of extracts from three extraction methods by capillary electrophoresis (Agilent 2100 Bioanalyzer); and (4) formulate a practical decision framework incorporating cost‐per‐successful‐PCR‐outcome analysis.

## 2 Materials and Methods

### 2.1 Study Organisms and Specimen Preservation

*Vertigo antivertigo* (Draparnaud, 1801) served as the primary model species for method comparison owing to its abundance in Lithuanian wetland habitats. Adults (shell height 2.0–2.5 mm) were collected by hand‐sorting moss and litter from fen and marsh habitats across Lithuania. Specimens originated from collections made between 2008 and 2025. Species determination was performed in the field and laboratory by an experienced malacologist. Specimens were preserved in 96% ethanol in 2 mL microtubes and stored at room temperature until extraction. To minimise confounding by specimen age or preservation history, the 60 specimens used in the main comparison were drawn from collections spanning 2008–2025 and randomly allocated to the six extraction methods (*n* = 10 per method) using a block‐randomised design stratified by collection period, where specimens were grouped into approximately 5‐year intervals based on label data and every method received an identical allocation across those periods: three specimens from the oldest period (2008–2012), two from each of the two intermediate periods (2013–2017 and 2018–2022) and three from the most recent (2023–2025), a 3:2:2:3 distribution giving *n* = 10 per method and 60 specimens in total. The full allocation is given in Table S3. Ethanol was not refreshed after initial preservation; replacement frequency and precise storage histories were not systematically recorded, and potential confounding by specimen age therefore could not be fully disentangled (see Section 4.4).

Specimens of *V*. *antivertigo* were collected from non‐protected habitats where no specific collection permit was required under Lithuanian law. The three EU Habitats Directive Annex II species (*V*. *angustior*, *V*. *geyeri*, *V*. *moulinsiana*) used for sequencing verification were obtained from the collection of Vilnius University Museum of Zoology, originally collected under permit No. 600000‐S‐55 (2020‐06‐08) issued by the Lithuanian Environmental Protection Agency (Aplinkos apsaugos agentūra).

### 2.2 DNA Extraction Protocols

Six methods were compared (Table 1; full step‐by‐step protocols in Table S1): alkaline lysis (HotSHOT destructive and non‐destructive variants), organic extraction (modified CTAB + PVP‐40), and three commercial silica‐column kits. Ten specimens of *V*. *antivertigo* were processed individually per method (*n* = 10 per method). A negative extraction control (reagents only, no tissue) accompanied each batch. Because some destructive HotSHOT extracts showed weak or inconsistent amplification during preliminary screening—a pattern consistent with carry‐over inhibition rather than insufficient template—a separate pilot optimisation assay was subsequently conducted on an additional set of destructive HotSHOT extracts (see Section  2.4); those results are reported independently from the main six‐method comparison.

**TABLE 1:** Six DNA extraction methods compared, with estimated bench time (excluding overnight digestion where applicable) and reagent cost per sample.

Method	Category	Key features	Elution (μL)	Time (h)	Cost/sample (EUR)	Voucher
HotSHOT (destructive)	Alkaline lysis	Whole individual (soft body plus shell) crushed; 25 mM NaOH → Tris–HCl (pH 8.0); crude lysate	50	~0.7	< 0.10	No
HotSHOT (non‐destructive)	Alkaline lysis	Intact individual (soft body plus shell) placed in 25 mM NaOH at 95°C without crushing; shell rinsed with 96% ethanol and retained as a morphological voucher	50	~0.7	< 0.10	**Yes**
CTAB + PVP‐40	Organic extraction	Whole individual (soft body plus shell) manually disrupted; 2% CTAB +1% PVP‐40 + DTT; phenol:chloroform:isoamyl alcohol (25:24:1) cleanup; isopropanol precipitation with glycogen; 75% ethanol wash; resuspension in TE	15–20	~3–4	~0.70	No
GeneJET Genomic DNA Purification Kit (Thermo Fisher Scientific)	Silica column	Whole individual (soft body plus shell) crushed; kit lysis buffer + proteinase K; overnight digestion at 56°C	100	~2–3	~2.50	No
DNeasy Blood & Tissue Kit (Qiagen)	Silica column	Whole individual (soft body plus shell) crushed; Buffer ATL + proteinase K; overnight digestion at 56°C; dual elution with Buffer AE at 70°C	100	~2–3	~3.50	No
QIAamp DNA Micro Kit (Qiagen)	Microcolumn	Whole individual (soft body plus shell) crushed; kit lysis + carrier RNA; overnight digestion at 56°C; low‐volume elution with Buffer AE at 70°C	20–30	~2–3	~5.00	No

*Note:* Costs reflect reagent prices as of 2024–2025; consumables excluded. Bold ‘Yes’ denotes the only method that retains the shell as a morphological voucher.

Abbreviations: CTAB, hexadecyltrimethylammonium bromide; DTT, dithiothreitol; PVP‐40 = polyvinylpyrrolidone.

#### 2.2.1 HotSHOT Protocol—Destructive Variant

Specimens were removed from ethanol, air‐dried for 5 min and shells crushed in 0.2 mL PCR tubes using single‐use sterile micropestles (one per specimen) to prevent cross‐contamination; the work surface was decontaminated with 10% sodium hypochlorite between samples. Crushed material was incubated in 50 μL of 25 mM NaOH at 95°C for 30 min in a thermocycler. Following cooling to 10°C, 5 μL of 1 M Tris–HCl (pH 8.0) was added to neutralise the lysate. The lysate was centrifuged (14,000*g*, 10 min) and the supernatant used directly or stored at −20°C (Truett et al. 2000).

#### 2.2.2 HotSHOT Protocol—Non‐Destructive (Shell‐Preserving) Variant

Intact individuals (snail within shell) were transferred to 0.2 mL PCR tubes containing 50 μL of 25 mM NaOH and incubated at 95°C for 30 min. The lysate was neutralised as above. The emptied shell was then recovered from the lysate under a stereomicroscope using fine sterile forceps (Dumont No. 5 or equivalent, ethanol‐flame‐sterilised between specimens). To avoid mechanical damage to the alkaline‐weakened shell, the forceps were positioned beneath the aperture to cradle rather than grip the shell; alternatively, when the shell was exceptionally fragile, the lysate was carefully aspirated from the tube using a micropipette set to 50 μL, leaving the shell undisturbed at the bottom, after which the shell was gently tipped onto a clean glass slide. The recovered shell was rinsed twice with 96% ethanol to remove residual NaOH, air‐dried at room temperature, and retained as a morphological voucher. Because alkaline heat exposure may partially degrade the shell's organic periostracum and conchiolin matrix, recovered shells were handled with particular care.

#### 2.2.3 Shell Voucher Assessment

Shells recovered from the non‐destructive HotSHOT protocol were examined under a stereomicroscope for structural integrity (intact, cracked, or fragmented) and visibility of taxonomically diagnostic aperture dentition.

#### 2.2.4 Modified CTAB Protocol With PVP‐40

Total genomic DNA was extracted using a modified CTAB protocol (adapted from Chakraborty et al. 2020). Because the soft body (< 1 mg) cannot be separated from the shell at this body size without unacceptable tissue loss, the whole individual—soft body together with the shell—served as extraction input. Briefly, the whole specimen was mechanically homogenised using a sterile micropestle in 200 μL of CTAB extraction buffer (2% w/v CTAB, 100 mM Tris–HCl pH 8.0, 1.4 M NaCl, 20 mM EDTA, 1% w/v PVP‐40). PVP‐40 was included to sequester polyphenolic compounds and glycosaminoglycans that co‐purify with DNA in gastropod tissues, as established for plant and mollusc DNA extraction (Porebski et al. 1997; Chakraborty et al. 2020). To mitigate nucleic acid degradation and neutralise nucleases, the buffer was supplemented with 50 μL of 0.1 M dithiothreitol (DTT) (yielding a final concentration of approximately 20 mM). This elevated DTT concentration, relative to conventional 2 mM formulations, was adopted to ensure complete reduction of disulfide‐linked mucus glycoproteins under the high‐polysaccharide lysis conditions characteristic of gastropod soft tissue. The buffer was further supplemented with 5 μL of proteinase K (20 mg/mL). The homogenate was incubated at 60°C for 2 h with continuous agitation at 800 rpm in a thermomixer.

Following lysis, an equal volume (~250 μL) of phenol:chloroform:isoamyl alcohol (25:24:1) was added. To prevent DNA shearing, the mixture was gently inverted for 5–10 min instead of vortexing, followed by centrifugation at 12,000*g* for 15 min at room temperature. The upper aqueous phase was carefully recovered, and DNA was precipitated by adding 0.1 volumes of 3 M sodium acetate (pH 5.2), 0.6 volumes of cold isopropanol and 1 μL of glycogen (20 mg/mL) as a carrier. The mixture was incubated at −20°C for ≥ 1 h and centrifuged at 12,000*g* for 30 min at 4°C. The resulting pellet was washed with 500 μL of cold 75% ethanol, air‐dried for 5 min until translucent and slowly resuspended in 15–20 μL of TE buffer overnight at 4°C.

#### 2.2.5 Commercial Silica‐Column Kits

##### 2.2.5.1 GeneJET Genomic DNA Purification Kit (Thermo Fisher Scientific)

Manufacturer's tissue protocol; overnight proteinase K digestion at 56°C; whole individuals (soft body plus shell) crushed prior to lysis. Elution volume: 100 μL.

##### 2.2.5.2 DNeasy Blood & Tissue Kit (Qiagen)

Manufacturer's tissue protocol; overnight lysis in Buffer ATL at 56°C; whole individuals (soft body plus shell) crushed. Elution: 2 × 50 μL Buffer AE at 70°C.

##### 2.2.5.3 QIAamp DNA Micro Kit (Qiagen)

Manufacturer's tissue protocol; whole individuals (soft body plus shell) crushed; overnight lysis at 56°C; carrier RNA added per manufacturer's recommendation. Elution: 20–30 μL Buffer AE at 70°C. Both Qiagen kits were included because they are designed for different input regimes rather than because of the manufacturer: DNeasy Blood & Tissue is a general‐purpose tissue kit optimised for milligramme‐scale input with a large elution volume, whereas QIAamp DNA Micro is formulated for trace‐scale samples, using carrier RNA to improve recovery and a low elution volume to concentrate the eluate. Because sub‐milligramme input is the defining constraint in Vertiginidae, comparing a standard tissue kit against a low‐input kit built on the same silica‐column chemistry was one of the questions this study set out to answer.

### 2.3 DNA Quantification and Integrity Assessment

DNA concentration was measured by QuantiFluor dsDNA fluorometry (Promega) and NanoDrop 2000 spectrophotometry (Thermo Fisher Scientific; A260/280 and A260/230 ratios). The six‐method comparison on *V. antivertigo* (*n* = 10 per method) was evaluated by QuantiFluor fluorometry and NanoDrop spectrophotometry. Capillary electrophoresis was performed on a separate specimen set and for three methods only (see below), and therefore does not contribute to the six‐method comparison. To characterise fragment‐size distributions for three extraction methods (DNeasy Blood & Tissue, CTAB + PVP‐40 and destructive HotSHOT), a separate set of 10 individuals of *V*. *antivertigo* (distinct from the 10 specimens per method used in the main comparison) was extracted with each method and assessed by capillary electrophoresis on an Agilent 2100 Bioanalyzer using the High Sensitivity DNA Assay. A separate specimen set was used because the assay consumes 1 μL of eluate, a material fraction of these low‐volume extracts, which would have compromised the PCR series for which the main‐comparison extracts were reserved. Profiling was restricted to three methods because they represent the three mechanistically distinct extraction classes compared here—silica‐column purification, organic extraction and alkaline lysis—and because extending it to the remaining three would have required destroying a further 30 specimens for a measurement that is descriptive rather than inferential in this design. Fragment‐size distributions were summarised in the 200–1000 bp window because this interval spans the targeted amplicon lengths, although fragments longer than 1000 bp may also remain PCR‐competent. The concentration, corrected area and percentage of total signal within this selected window were recorded for each sample (Table 2). Between‐method differences were evaluated separately for each metric using the Kruskal‐Wallis test. Bonferroni‐adjusted pairwise Mann–Whitney *U* comparisons were computed for the percentage of total signal within the selected window only, and pairwise significance is therefore reported for that metric alone.

**TABLE 2:** Fragment‐size distribution in the selected 200–1000 bp window for DNeasy Blood & Tissue, CTAB + PVP‐40 and destructive HotSHOT extracts of *Vertigo antivertigo* (96% ethanol; *n* = 10 independently extracted individuals per method; Agilent 2100 Bioanalyzer, High Sensitivity DNA Assay).

Method	Corr. area	% of total	Conc. (pg/μL)	Avg. size (bp)
DNeasy Blood & Tissue	268.9 (247.4–329.3)	42 (34–52)	730 (454–1515)	476 (465–499)
CTAB + PVP‐40	54.7 (36.2–72.3)	21 (13–33)	37 (17–54)	463 (383–514)
HotSHOT (destructive)	7.5 (2.5–10.9)	8 (4–15)	8 (3–19)	694 (615–791)
Kruskal–Wallis *p*	< 0.001	< 0.001	< 0.001	0.013

*Note:* Values are median (IQR). This window spans the targeted amplicon lengths but should not be interpreted as a direct measure of total amplifiable DNA abundance. Summary data underlying this table are available at Zenodo (https://doi.org/10.5281/zenodo.21984159).

### 2.4 PCR Amplification and Sequencing

PCR was carried out in 25 μL reactions using Platinum II Hot‐Start PCR Master Mix (2×; Thermo Fisher Scientific). Each reaction contained 12.5 μL of master mix and 0.4 μM of each primer. Template DNA (1 μL undiluted lysate or eluate) was added per reaction. A fixed template volume rather than a normalised template mass was used because the comparison asks which method delivers a usable PCR result from a single specimen: the shell‐preserving protocol yielded a median of 0.17 ng/μL, so a normalised input of tens of nanograms is unattainable from one *Vertigo* individual, and normalisation would have excluded the very method this study exists to evaluate. Four loci were targeted: COI barcode (~658 bp; LCO1490/HCO2198; Folmer et al. 1994), COI mini‐barcode (~313 bp; mlCOIintF, Leray et al. 2013/jgHCO2198, Geller et al. 2013), ITS1 (~600 bp; ITS1/ITS2; White et al. 1990), and ITS2 (~450–600 bp; ITS3/ITS4; White et al. 1990). Primer sequences, source references and expected amplicon lengths are provided in the Supporting Information (Table S2). Each extract was initially screened under standard PCR conditions without additives. Thermal cycling conditions were locus‐specific. For COI and COI mini‐barcode: initial denaturation at 98°C for 3 min; 35 cycles of 98°C for 10 s, 48°C for 35 s and 68°C for 35 s; final extension at 68°C for 5 min. For ITS1 and ITS2: initial denaturation at 98°C for 3 min; 35 cycles of 98°C for 10 s, 51°C for 35 s and 68°C for 35 s; final extension at 68°C for 5 min. Because some destructive HotSHOT extracts showed weak or inconsistent amplification during preliminary screening, a separate pilot follow‐up assay was performed on an additional set of such extracts, analysed independently from the 10 specimens summarised in Table 3. In that assay, each extract was tested under standard conditions and, in parallel, with additive supplementation: each 25 μL reaction was supplemented with 0.5 μL bovine serum albumin (BSA; 20 mg/mL stock, final concentration 0.4 mg/mL; Kreader 1996) and 2.5 μL of a 10% trehalose solution (final concentration 1%; Spiess et al. 2004; Samarakoon et al. 2013). All four loci were amplified per extract in both conditions. No‐template negative controls were included in every run. Amplification success was scored by the presence of a band of expected size on an agarose gel (1.5% agarose in 1× TAE; stained with ethidium bromide; visualised under UV; fragment size estimated using FastRuler Low Range DNA Ladder, Thermo Fisher Scientific). A subset of 12 representative successful amplicons (3 methods × 4 loci: DNeasy Blood & Tissue, CTAB and non‐destructive HotSHOT) was Sanger‐sequenced bidirectionally (ABI 3730xl) solely to confirm amplicon identity—that is, to verify that the bands scored on the gel were the intended targets rather than primer artefacts or non‐target amplification. Paired reads were assembled in Geneious Prime 2026.0; consensus sequences were accepted when the mean Phred quality score reached ≥ 20 across at least 80% of base positions.

**TABLE 3:** PCR amplification success (successes/*n*) for four loci across six extraction methods applied to *Vertigo antivertigo* preserved in 96% ethanol.

Method	COI 658 bp	COI mini 313 bp	ITS1	ITS2	Overall PCR (*n*/40, %)
HotSHOT (destructive)	10/10	10/10	10/10	10/10	40/40 (100)
HotSHOT (non‐destructive)	8/10	10/10	9/10	9/10	36/40 (90)
CTAB + PVP‐40	10/10	10/10	10/10	10/10	40/40 (100)
GeneJET Genomic	6/10	8/10	7/10	6/10	27/40 (67.5)
DNeasy Blood & Tissue	10/10	10/10	10/10	10/10	40/40 (100)
QIAamp DNA Micro	10/10	10/10	10/10	10/10	40/40 (100)

*Note:* PCR was performed under standard conditions (undiluted templates, no additives). Overall PCR (n/40, %) denotes total successful reactions across the four loci. The separate HotSHOT rescue assay with BSA + trehalose is reported in the main text and is not included in this baseline comparison.

### 2.5 Sequence Verification on EU Habitats Directive Annex II Species

Separately from the amplicon‐identity check described above, ITS1 amplicons from three *Vertigo* species listed in Annex II of the EU Habitats Directive—*V. angustior* (sample BJ‐2), *V. geyeri* (BJ‐3) and *V. moulinsiana* (BJ‐4)—were Sanger‐sequenced bidirectionally (ABI 3730xl). These are the taxa for which a shell‐preserving protocol is actually needed, and the purpose of this step was to test whether a workflow developed on the abundant, unprotected *V. antivertigo* transfers to the protected species for which destructive sampling is least acceptable. All three specimens were extracted with the shell‐preserving non‐destructive HotSHOT protocol. ITS1 was selected because it amplified most consistently across our material and because it has the densest reference coverage for European *Vertigo* in GenBank (Nekola et al. 2018), which makes species‐level assignment by BLASTn interpretable. Reads were assessed for ambiguous base calls (N or IUPAC codes), for secondary chromatogram peaks exceeding 30% of the primary peak height at bases called at *Q* ≥ 20, and for forward–reverse concordance over the aligned overlap; for this assessment all reads were quality‐trimmed to the first and last base at *Q* ≥ 20.

### 2.6 Statistical Analysis

All analyses were conducted in R v4.5.2. Continuous outcomes were summarised as median (IQR), and PCR amplification was summarised as successes/n with exact 95% Clopper‐Pearson confidence intervals (Clopper and Pearson 1934). Because several methods × locus combinations showed ceiling effects, emphasis in the present manuscript is on descriptive comparison rather than exhaustive hypothesis testing. Kruskal‐Wallis tests followed by pairwise Mann–Whitney *U* comparisons with Bonferroni correction were used for exploratory comparisons of concentration/purity where informative. For the Bioanalyzer fragment‐profile comparison (DNeasy Blood & Tissue, CTAB + PVP‐40 and destructive HotSHOT; *n* = 10 per method), between‐method differences in the percentage of total signal within the selected 200–1000 bp window were evaluated using the Kruskal‐Wallis test followed by pairwise Mann–Whitney *U* comparisons (Bonferroni‐adjusted). Cost per successful COI PCR outcome was calculated as extraction‐plus‐PCR cost (reagent prices as of 2024–2025) divided by the observed full‐length COI success proportion, with a ±20% sensitivity analysis.

## 3 Results

### 3.1 DNA Concentration and Purity

**FIGURE 1:**
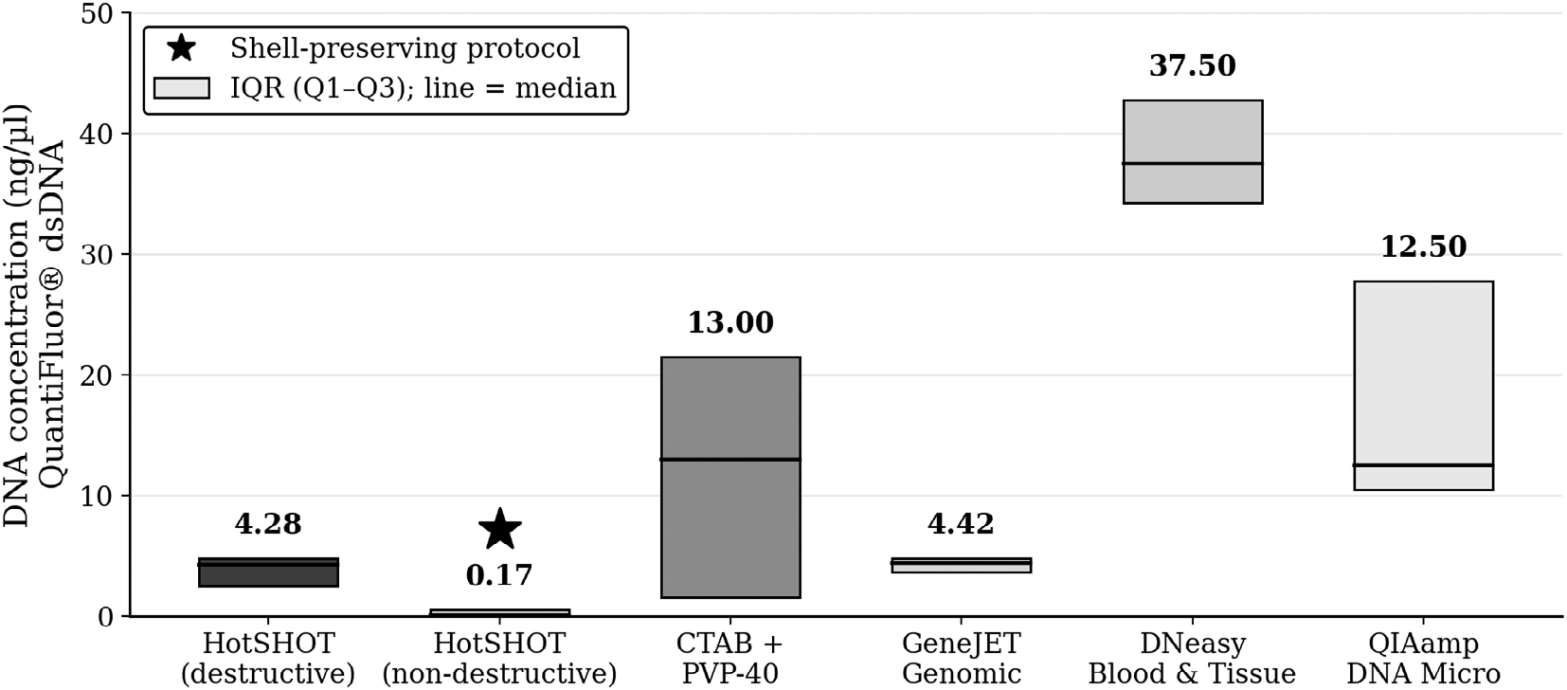
DNA concentration (QuantiFluor dsDNA) for six extraction methods applied to *Vertigo antivertigo* preserved in 96% ethanol. Values indicate medians and shaded boxes indicate interquartile ranges (*n* = 10 per method). The star marks the shell‐preserving non‐destructive HotSHOT protocol.

**FIGURE 2:**
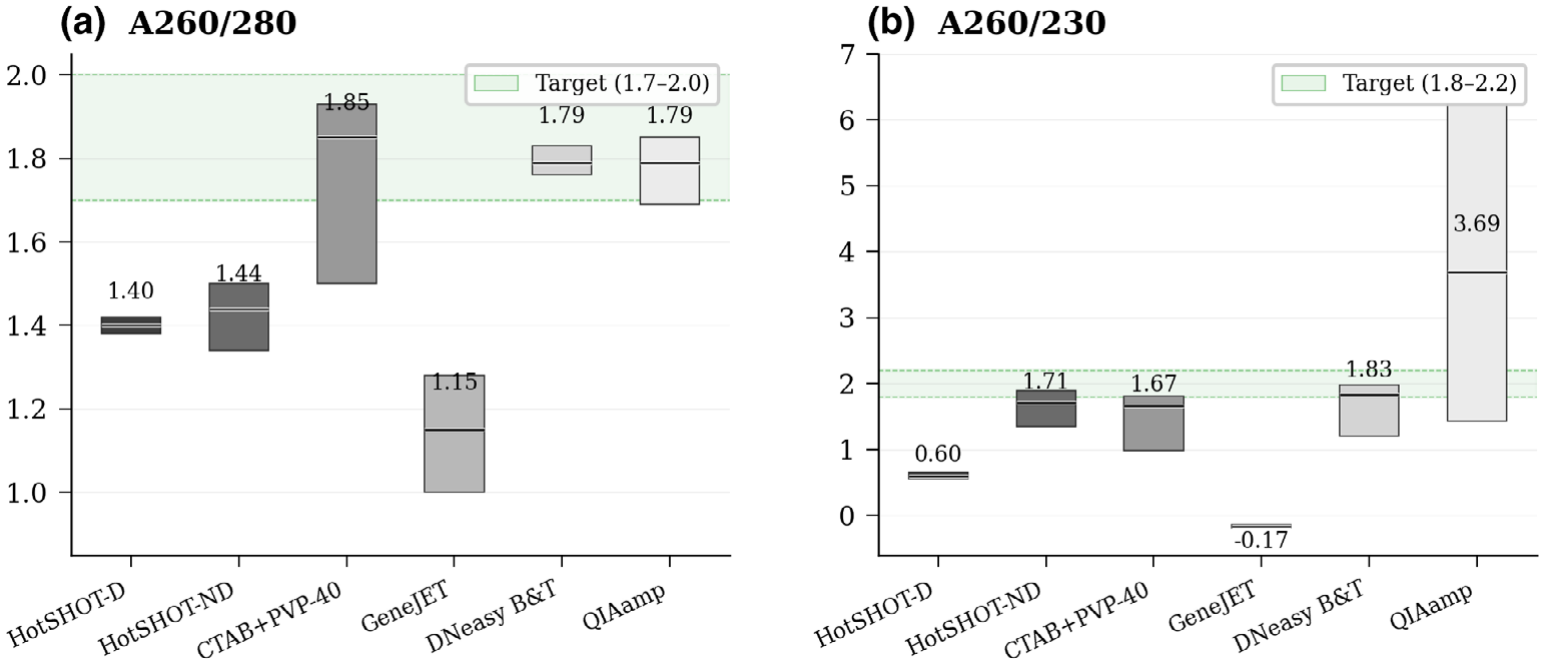
Spectrophotometric purity (NanoDrop A260/280 and A260/230 ratios) for six extraction methods applied to *Vertigo antivertigo* preserved in 96% ethanol. Boxes show interquartile ranges; values denote medians; green bands indicate target purity windows (*n* = 10 per method).

DNA concentration and spectrophotometric purity for all six methods are provided in Table 4. NanoDrop data revealed pronounced differences between the two HotSHOT variants: destructive extracts exhibited low A260/230 ratios (median 0.60, IQR 0.56–0.66; *n* = 10), indicating substantial carry‐over of non‐DNA absorbers, whereas non‐destructive extracts showed markedly improved A260/230 values (median 1.71, IQR 1.35–1.89; *n* = 10), pointing to reduced contaminant transfer during the shell‐recovery step. A260/280 ratios were similar between variants (destructive: median 1.40, IQR 1.38–1.42; non‐destructive: median 1.44, IQR 1.34–1.50), but fluorometric concentration was much lower for the shell‐preserving protocol (median 0.17 ng/μL, IQR 0.08–0.57) than for destructive HotSHOT (median 4.28 ng/μL, IQR 2.51–4.83). NanoDrop‐derived concentration readings were not retained in the archived dataset and therefore cannot be compared quantitatively with the fluorometric measurements. We consequently report fluorometric concentrations together with the retained A260/280 and A260/230 absorbance ratios, without inferring a concentration discrepancy between instruments. At the sub‐nanogram‐per‐microlitre concentrations typical of shell‐preserving extracts, fluorometric quantification should be treated as the reference method for low‐input Vertiginidae extracts (Figures 1 and 2).

**TABLE 4:** DNA concentration (QuantiFluor dsDNA) and spectrophotometric purity (NanoDrop A260/280 and A260/230) for six extraction methods applied to *Vertigo antivertigo* preserved in 96% ethanol (*n* = 10 per method).

Method	Concentration (ng/μL)	A260/280	A260/230	Voucher	Total DNA per specimen (ng)
HotSHOT (destructive)	4.28 (2.51–4.83)	1.40 (1.38–1.42)	0.60 (0.56–0.66)	No	214
HotSHOT (non‐destructive)	0.17 (0.08–0.57)	1.44 (1.34–1.50)	1.71 (1.35–1.89)	Yes	8.5
CTAB + PVP‐40	13.00 (1.59–21.50)	1.85 (1.50–1.93)	1.67 (0.98–1.81)	No	195–260
GeneJET Genomic	4.42 (3.65–4.80)	1.15 (1.00–1.28)	−0.17 (−0.19 to −0.14)	No	442
DNeasy Blood & Tissue	37.50 (34.25–42.75)	1.79 (1.76–1.83)	1.83 (1.20–1.98)	No	3750
QIAamp DNA Micro	12.50 (10.50–27.75)	1.79 (1.69–1.85)	3.69 (1.44–6.25)	No	250–375

*Note:* Values are median (IQR). Because elution volumes differ across methods (Table 1), concentrations are not directly comparable between methods; total DNA per specimen, obtained as median concentration × nominal elution volume, is given in the final column as an approximate basis for comparison. For QIAamp DNA Micro, A260/230 is based on *n* = 7 after exclusion of three samples with unstable NanoDrop baselines (see Section 3).

**FIGURE 3:**
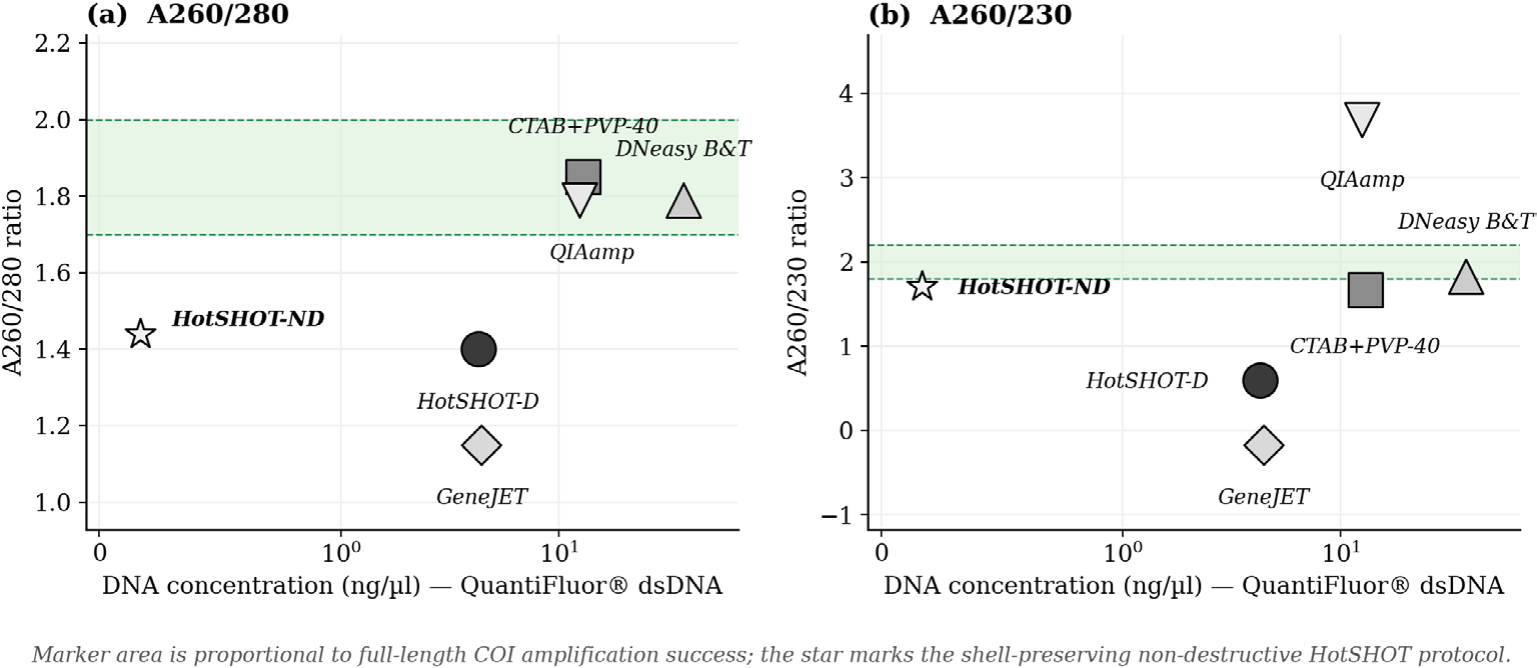
Concentration‐purity overview of six extraction methods applied to *Vertigo antivertigo*. (a) A260/280 and (b) A260/230 plotted against DNA concentration (QuantiFluor dsDNA). Bubble area is proportional to full‐length COI amplification success; star indicates the shell‐preserving non‐destructive HotSHOT protocol. Green bands mark the ideal ranges (A260/280 1.7–2.0; A260/230 1.8–2.2).

Among the remaining methods, DNeasy Blood & Tissue produced the highest fluorometric concentrations (median 37.50 ng/μL, IQR 34.25–42.75) together with near‐ideal A260/280 (median 1.79) and acceptable A260/230 values (median 1.83, IQR 1.20–1.98). CTAB + PVP‐40 and QIAamp DNA Micro yielded intermediate extract concentrations (medians 13.00 and 12.50 ng/μL, respectively). CTAB extracts showed the highest A260/280 ratio (median 1.85) and moderately clean A260/230 values (median 1.67), whereas QIAamp DNA Micro extracts combined good A260/280 with highly variable A260/230 values (median 3.69, IQR 1.44–6.25; *n* = 7 after exclusion of three samples where instrument noise around the 230 nm absorbance trough produced unstable baselines, likely attributable to near‐zero absorbance signals in these low‐input eluates; all three affected samples retained valid fluorometric concentration values). In this low‐input context, the QIAamp DNA Micro purity ratios should be interpreted cautiously because weak absorbance signals and carrier RNA may affect NanoDrop‐based estimates. GeneJET Genomic extracts performed least well by purity metrics, with low A260/280 (median 1.15) and near‐zero or negative A260/230 values (median −0.17), indicating poor signal quality and/or persistent contaminants (Figure 3).

### 3.2 Fragment‐Size Distribution (Bioanalyzer)

**FIGURE 4:**
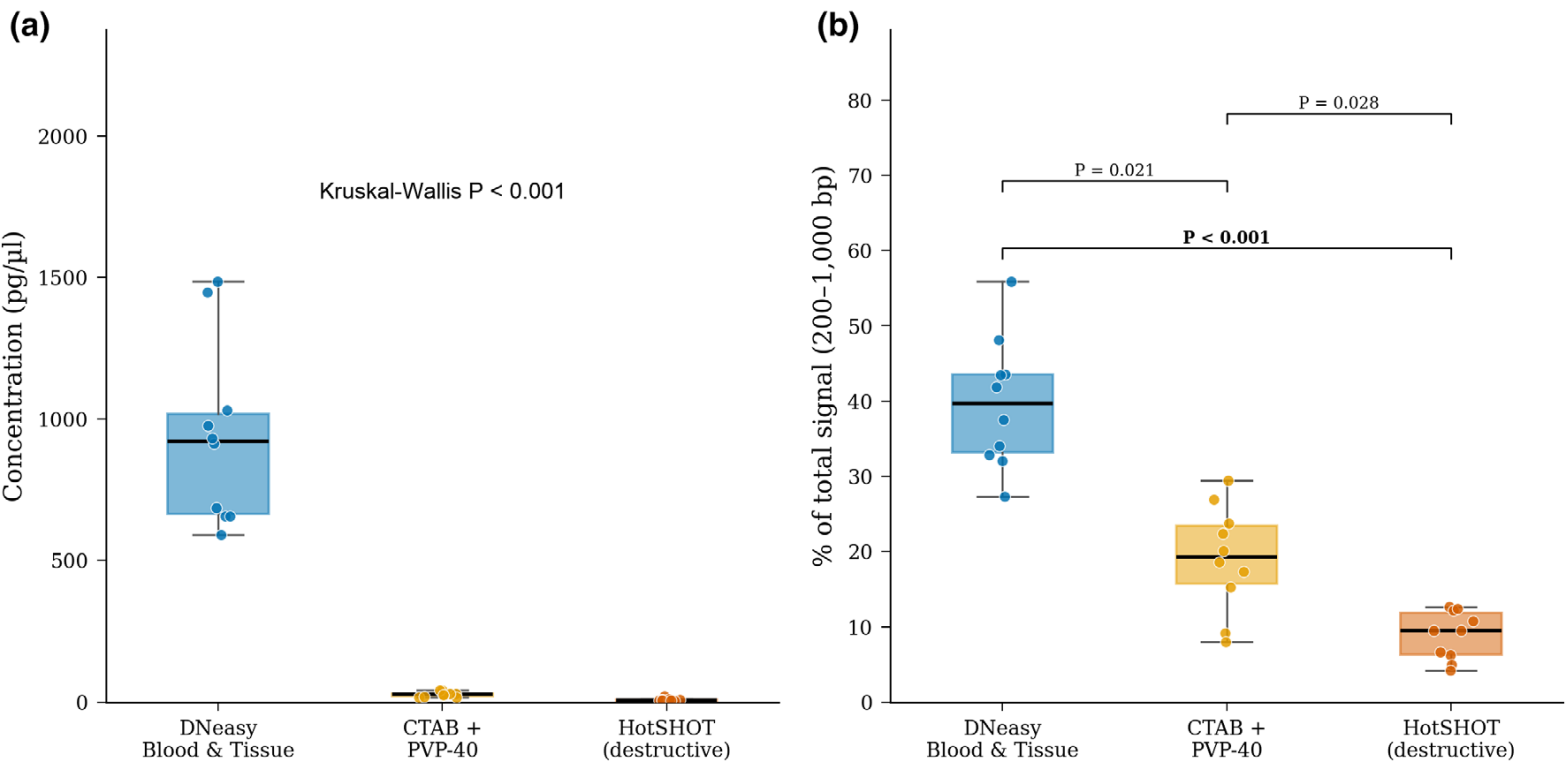
Fragment‐size distribution (200–1000 bp window) for DNeasy Blood & Tissue, CTAB + PVP‐40 and destructive HotSHOT extracts of *Vertigo antivertigo* (*n* = 10 per method; Bioanalyzer High Sensitivity DNA Assay). (a) Concentration. (b) Percentage of total signal. Boxes = IQR; lines = median. Brackets in (b) indicate Bonferroni‐adjusted pairwise Mann–Whitney *U*, *p* values; pairwise comparisons were computed for the percentage‐of‐signal metric only. The *p* value shown in (a) is the Kruskal–Wallis result for concentration.

Capillary electrophoresis on the Agilent 2100 Bioanalyzer (High Sensitivity DNA Assay) revealed pronounced differences in fragment‐size distribution among the three profiled extraction methods (Table 2; Figure 4; *n* = 10 independently extracted individuals per method, distinct from the main comparison set). In the selected 200–1000 bp window, DNeasy Blood & Tissue extracts contained a median of 42% (IQR 34%–52%) of total electrophoretic signal, corresponding to a median concentration of 730 pg/μL (IQR 454–1515). CTAB + PVP‐40 extracts showed an intermediate profile, with a median of 21% (IQR 13%–33%) and a median concentration of 37 pg/μL (IQR 17–54). By contrast, destructive HotSHOT extracts showed a median of only 8% (IQR 4%–15%) of total signal in this region, with a median concentration of 8 pg/μL (IQR 3–19). Between‐method differences in the percentage of total signal were significant (Kruskal‐Wallis H = 15.82, *p* < 0.001), with all Bonferroni‐adjusted pairwise Mann–Whitney *U* comparisons reaching significance (DNeasy Blood & Tissue vs. HotSHOT: *p* < 0.001; DNeasy Blood & Tissue vs. CTAB: *p* = 0.021; CTAB vs. HotSHOT: *p* = 0.028). Kruskal‐Wallis tests on the remaining Bioanalyzer metrics were likewise significant (concentration and corrected area, both *p* < 0.001; average fragment size, *p* = 0.013; Table 2); pairwise comparisons were not computed for those metrics, and the pairwise *p* values above therefore apply to the percentage‐of‐signal metric only. Several HotSHOT samples yielded no detectable signal within the 200–1000 bp window. These data indicate that destructive HotSHOT lysates contained proportionally less signal in the selected 200–1000 bp window than DNeasy Blood & Tissue or CTAB extracts. Because fragments longer than 1000 bp can also serve as PCR template for the loci targeted here, this metric is best interpreted as a comparative descriptor of fragment‐profile differences rather than a direct estimate of amplifiable template abundance.

### 3.3 PCR Amplification Success

**FIGURE 5:**
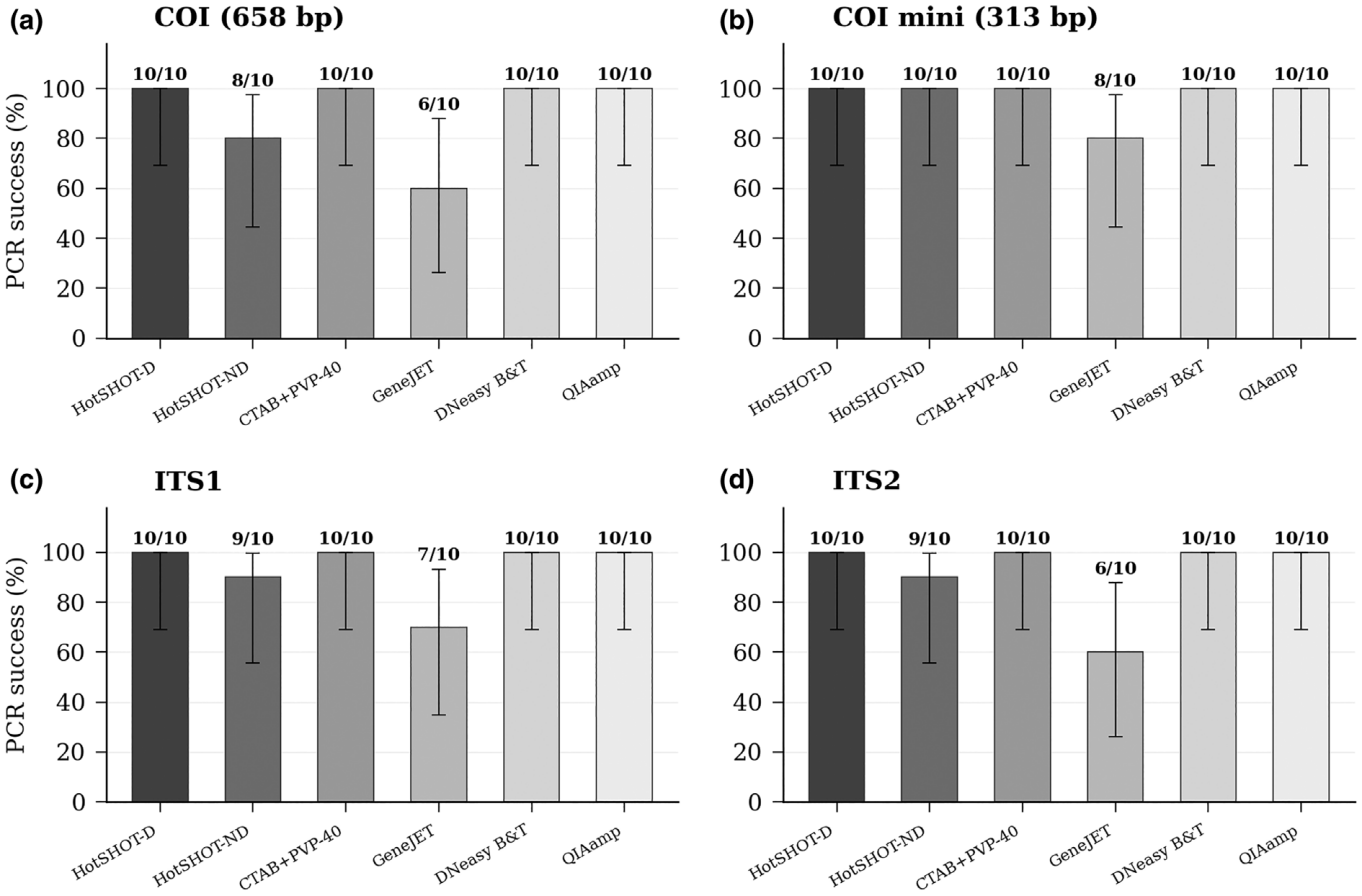
PCR success rates for four loci (COI, COI mini‐barcode, ITS1, ITS2) across six extraction methods under standard conditions (undiluted template, no additives). Points show observed success proportions; error bars show exact 95% Clopper‐Pearson binomial confidence intervals.

Table 3 summarises PCR success across four loci under the initial standard reaction conditions (*n* = 10 per method). Destructive HotSHOT, CTAB + PVP‐40, DNeasy Blood & Tissue and QIAamp DNA Micro each achieved 10/10 amplification success for all four loci. The shell‐preserving non‐destructive HotSHOT protocol remained highly effective, yielding 8/10 successful amplifications for full‐length COI, 10/10 for the COI mini‐barcode, and 9/10 for both ITS loci. GeneJET Genomic showed the weakest and least consistent amplification profile, with success rates of 6/10 for full‐length COI, 8/10 for the COI mini‐barcode, 7/10 for ITS1 and 6/10 for ITS2. As expected, the shorter COI mini‐barcode was at least as robust as the full‐length COI fragment across all methods (Figure 5). In a separate pilot follow‐up assay on a distinct batch of destructive HotSHOT extracts that had shown inconsistent amplification during preliminary screening, the standard‐condition overall success rate across all four loci was 70% (28/40 reactions across 10 extracts × 4 loci). Addition of BSA (final 0.4 mg/mL) and trehalose (final 1%) to the same extracts increased overall success to 100% (40/40), suggesting that carry‐over inhibition was an important contributor to failure in this selected subset. No amplification was detected in extraction blanks or no‐template PCR controls.

### 3.4 Shell Voucher Condition

**FIGURE 6:**
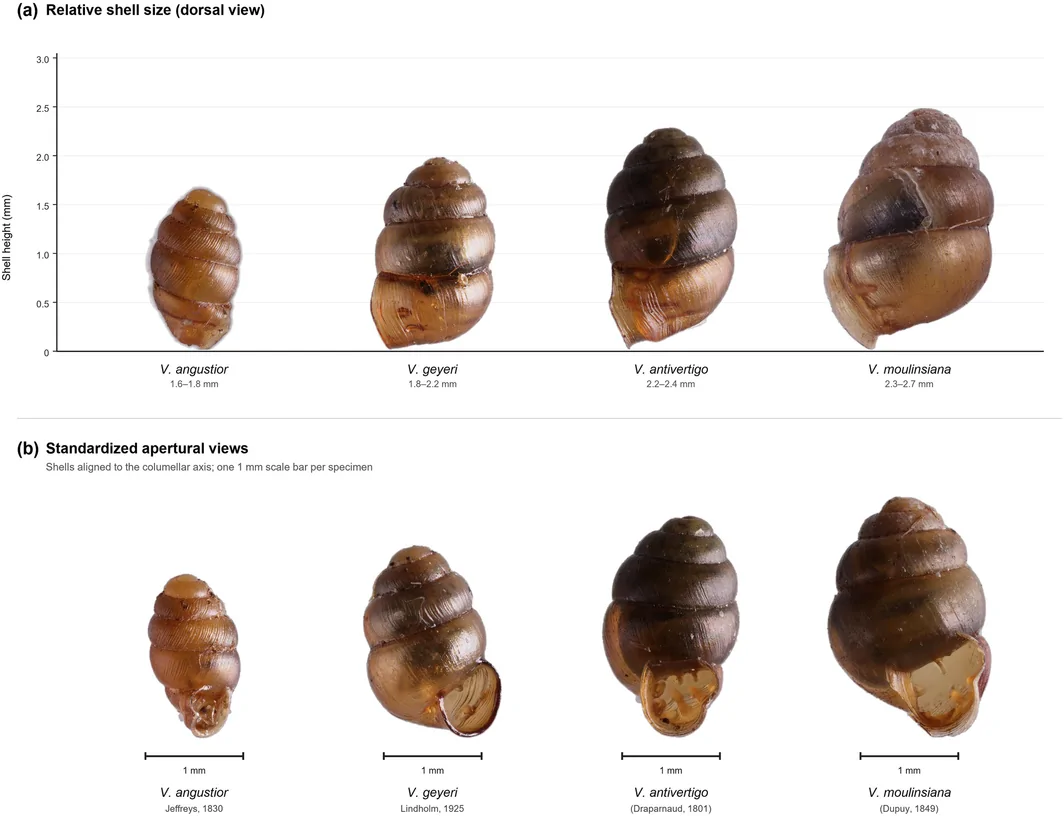
Shell morphology and relative size of the four *Vertigo* species included in this study. (a) Dorsal views scaled to a common height axis to show relative size. Reported adult shell‐height ranges are 1.6–1.8 mm for *V. angustior*, 1.8–2.2 mm for *V. geyeri*, 2.2–2.4 mm for *V. antivertigo* and 2.3–2.7 mm for *V. moulinsiana*. (b) Standardised apertural views aligned to the columellar axis, with one 1 mm scale bar per specimen. All panels use documented pre‐extraction photographs; the condition of the ten *V. antivertigo* vouchers recovered after non‐destructive HotSHOT extraction is reported in the Results.

All ten shells recovered from the non‐destructive HotSHOT protocol remained structurally intact (10/10); no cracking or fragmentation was observed. Apertural dentition, the primary diagnostic character for species‐level determination in Vertiginidae, was clearly visible under stereomicroscopy in all recovered specimens. Representative pre‐extraction shell morphology and relative size are shown in Figure 6. Shells were archived individually in 1.5 mL tubes as morphological vouchers (designated BJ‐1_Shell_voucher through BJ‐10_Shell_voucher). Alkaline heat exposure produced slight translucency of the periostracum in some specimens, but this did not impede taxonomic assessment.

### 3.5 Cost‐Effectiveness Based on COI PCR Success

**FIGURE 7:**
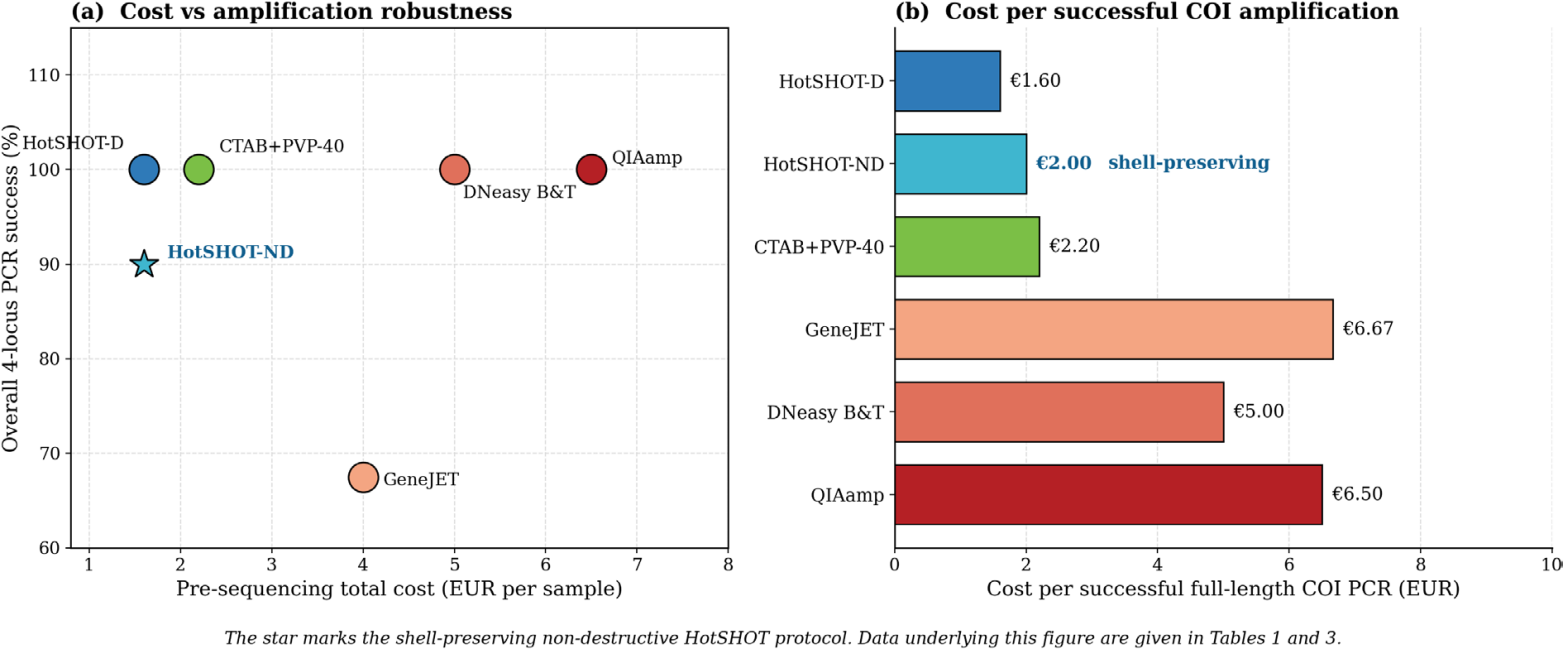
Cost‐effectiveness landscape of the six DNA extraction methods, showing pre‐sequencing total cost plotted against overall four‐locus PCR success (a) and cost per successful full‐length COI PCR (b), with the position of the shell‐preserving non‐destructive HotSHOT protocol indicated. Underlying values are given in Tables 1 and 3.

Pre‐sequencing cost‐effectiveness based on full‐length COI PCR success is summarised in Figure 7. Because reagent costs are listed in Table 1 and COI success proportions in Table 3, the figures below are derived quantities and are no longer tabulated separately. Under the observed success proportions, the lowest estimated pre‐sequencing cost per successful COI PCR result was obtained with destructive HotSHOT (€1.60), closely followed by the shell‐preserving non‐destructive HotSHOT (€2.00) and CTAB + PVP‐40 (€2.20). DNeasy Blood & Tissue and QIAamp DNA Micro traded higher per‐sample reagent costs for maximal amplification robustness (€5.00 and €6.50, respectively), whereas GeneJET Genomic was the least cost‐efficient option (€6.67), reflecting both its higher reagent cost and weaker amplification success. Sensitivity analysis (±20% change in success proportion) did not alter the overall ranking of the strongest performers.

### 3.6 Sanger Sequence Quality and Species Verification

Bidirectional ITS1 sequences were obtained for all three Annex II specimens (BJ‐2, *V. angustior*; BJ‐3, *V. geyeri*; BJ‐4, *V. moulinsiana*); the design and rationale are given in Materials and Methods.

**FIGURE 8:**
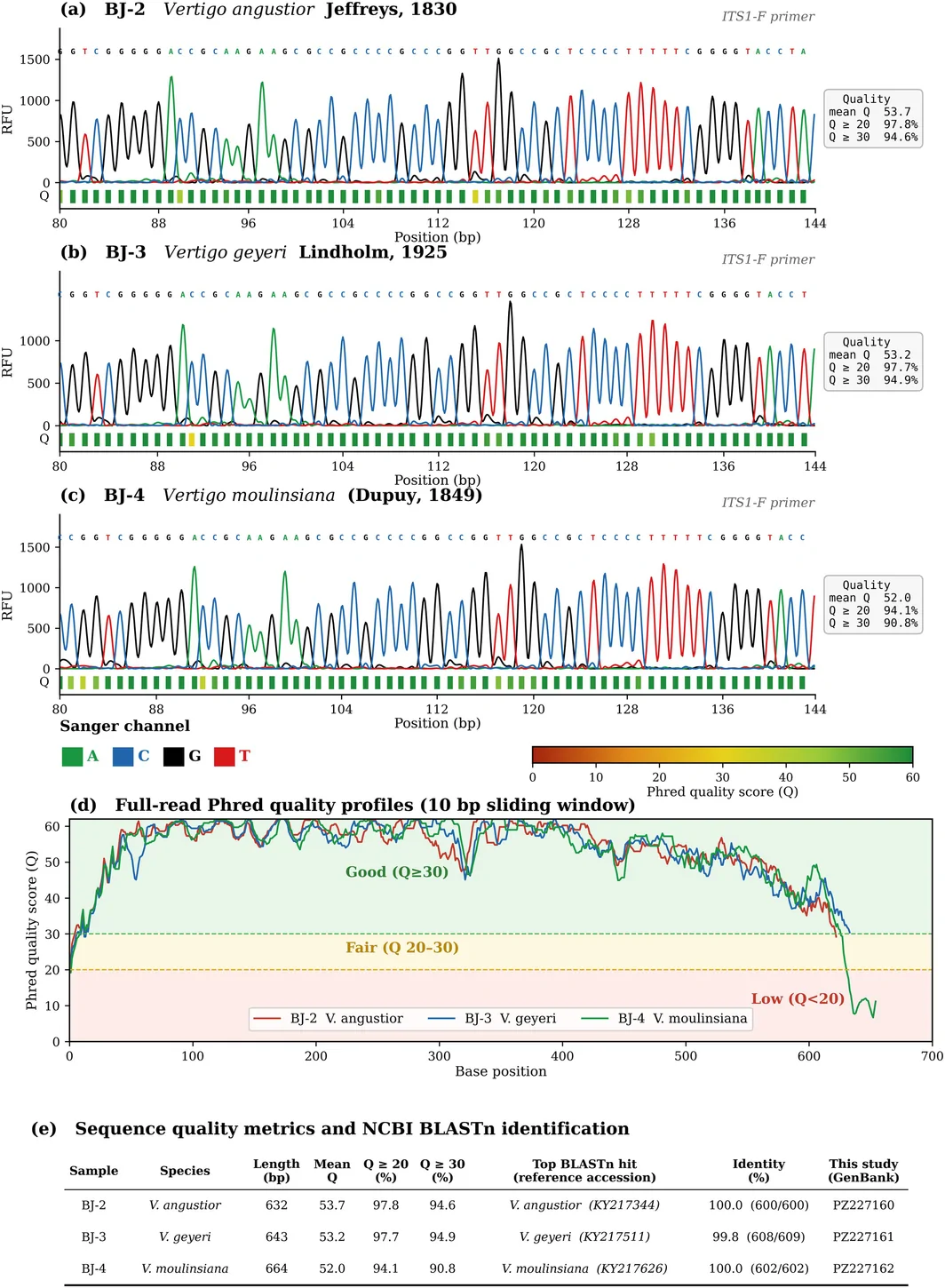
ITS1 Sanger sequence quality and NCBI BLASTn identification for three EU Habitats Directive Annex II *Vertigo* species extracted with the shell‐preserving non‐destructive HotSHOT protocol. (a–c) Representative Sanger chromatograms (forward reads, positions 80–144 bp) for (a) *Vertigo angustior* (BJ‐2); (b) *Vertigo geyeri* (BJ‐3); and (c) *Vertigo moulinsiana* (BJ‐4). Each chromatogram panel shows the four‐channel trace (A = green, C = blue, G = black, T = red), colour‐coded base calls above the peaks, a per‐base Phred quality heatmap strip (red‐yellow‐green; *Q* = 0–60), and summary statistics (mean *Q*; % bases with *Q* ≥ 20 and *Q* ≥ 30). (d) Full‐read Phred quality profiles (10 bp sliding‐window average) for all three samples; coloured zones indicate Good (*Q* ≥ 30), Fair (*Q* 20–30) and Low (*Q* < 20) quality regions. (e) Summary table of read length, mean Phred quality and percentage of bases at *Q* ≥ 20 and *Q* ≥ 30, together with the top NCBI BLASTn hit and its reference accession, the identity of that match, and the accession under which the sequence generated here has been deposited. Quality metrics are computed on reads trimmed to the first and last base at *Q* ≥ 20.

All three ITS1 forward reads produced clean, well‐resolved chromatograms with high signal‐to‐noise ratios across positions 80–144 bp (Figure 8a–c). After quality trimming to the first and last base at *Q* ≥ 20—a rule applied identically to all six reads reported here—mean Phred quality scores were 53.7 (BJ‐2), 53.2 (BJ‐3) and 52.0 (BJ‐4); the proportion of bases with *Q* ≥ 20 exceeded 94% in all three sequences, and *Q* ≥ 30 exceeded 90% (Figure 8d,e). NCBI BLASTn searches confirmed species‐level identifications: the ITS1 sequence of *V. angustior* (BJ‐2) matched GenBank accession KY217344 with 100% identity, *V. geyeri* (BJ‐3) matched KY217511 at 99.8% identity and *V. moulinsiana* (BJ‐4) matched KY217626 at 100% identity. For BJ‐3 the assignment rests on ITS1 alone: the CytB read obtained for this specimen was of insufficient quality to contribute (mean Phred *Q* 22.3, with 51% of bases at *Q* ≥ 20), and the specimen was independently determined as *V. geyeri* on shell morphology by an experienced malacologist. These results demonstrate that the extraction and PCR conditions evaluated for *V. antivertigo* are directly transferable to the protected *Vertigo* species that are the primary targets for conservation‐genetic applications of this workflow.

Reverse reads were comparable in quality to the forward reads: on the same trimming rule, mean Phred scores were 53.9 (BJ‐2), 52.6 (BJ‐3) and 53.9 (BJ‐4), with more than 94% of bases at *Q* ≥ 20 and more than 90% at *Q* ≥ 30 in every one of the six reads (Figure S1a–d,f). No ambiguous base calls (N or IUPAC codes) were present in any read. Among bases called at *Q* ≥ 20, positions at which the secondary chromatogram peak exceeded 30% of the primary peak height were rare (2–6 per read of approximately 630 bp, below 1%), and no such position was shared between the forward and the reverse read of the same specimen (Figure S1e); because a genuine heterozygous site necessarily produces a double peak in both directions, this pattern indicates residual trace noise rather than heterozygosity. Forward and reverse reads were 100.00% identical over the aligned overlap (602–610 bp) with no mismatches; the only differences were one to three single‐base indels at homopolymer runs near the read ends, which were resolved in the bidirectional consensus and do not represent frameshifts in the underlying sequence.

## 4 Discussion

The combined yield, purity, amplification and cost results identify three practical performance profiles. First, DNeasy Blood & Tissue provided the strongest all‐round performance for destructive extraction, combining the highest fluorometric extract concentration with uniformly successful amplification across all loci. QIAamp DNA Micro also amplified robustly across all targets, but achieved this at a substantially higher reagent cost and with more variable A260/230 values; in this low‐input context, those NanoDrop ratios should be interpreted cautiously. Second, CTAB + PVP‐40 offered a strong lower‐cost destructive alternative: although its fluorometric concentration was lower than DNeasy Blood & Tissue, purity metrics were acceptable and PCR performance was fully consistent across loci. This outcome aligns with the broader finding of Chakraborty et al. (2020) that modified CTAB yields superior reproducibility to commercial kits for non‐marine molluscs, though their study used substantially more tissue from larger species, and the advantage was less pronounced with our sub‐milligramme inputs. Third, the shell‐preserving non‐destructive HotSHOT protocol showed that very low measured extract concentrations do not necessarily preclude successful PCR in minute Vertiginidae, particularly for shorter targets. Its improved A260/230 profile relative to destructive HotSHOT is in line with reduced transfer of 230‐nm‐absorbing contaminants, even though absolute DNA recovery was much lower.

These results have direct implications for large‐scale *Vertigo* phylogenetics. Nekola et al. (2018) sacrificed all 424 shells in their global phylogenetic revision because no non‐destructive protocol was available for these minute specimens. Had a shell‐preserving extraction been feasible, the same individuals could have served both as DNA sources and as morphological vouchers, enabling direct genotype–phenotype comparisons within a genus where conchological convergence and plasticity confound taxonomic assignment (Nekola et al. 2018). We note that Nekola et al. (2018) documented their material photographically before extraction, so morphological information was not lost entirely, and photographic documentation should be standard practice before any destructive extraction. A photograph nevertheless cannot be re‐examined under different optics, measured in three dimensions, re‐imaged by scanning electron microscopy or micro‐CT, or serve as a physical reference specimen—which is precisely what a retained shell voucher provides. Our non‐destructive HotSHOT protocol, which retained all ten shells intact while yielding sequenceable DNA, demonstrates that such integrated molecular‐morphological workflows are now achievable for Vertiginidae.

Although this study used *V. antivertigo* as a model system, the challenges documented here—sub‐milligramme tissue input, mucus co‐purification and the need to retain voucher material for morphological verification—are broadly shared among minute terrestrial gastropods (shell height ≤ 5 mm) and, to varying degrees, among other small‐bodied invertebrates processed in large‐scale biodiversity surveys. Adema (2021) emphasised that the mucopolysaccharide problem in molluscs has no universal solution and must be addressed empirically for each tissue type and taxon; our data provide that empirical grounding for Vertiginidae. The performance profiles reported here therefore provide a practical decision framework applicable beyond Vertiginidae sensu stricto, including to museum and ethanol‐preserved reference collections where specimen material is scarce and shell vouchers retain scientific value (Jaksch et al. 2016; Goulding 2021).

The relationship between HotSHOT fragment profiles and PCR performance requires careful interpretation. In the primary six‐method comparison, destructive HotSHOT achieved 10/10 amplification success across all four loci. The Bioanalyzer and rescue experiments therefore should not be read as direct explanations of a poor baseline HotSHOT result in Table 3. Rather, they provide secondary evidence about failure modes that can arise in selected HotSHOT extracts. Specifically, destructive HotSHOT samples contributed less signal within the selected 200–1000 bp Bioanalyzer window than DNeasy Blood & Tissue or CTAB extracts, but that metric is not a direct measure of total amplifiable template. Likewise, the separate rescue assay shows that at least some initially problematic HotSHOT lysates respond strongly to BSA and trehalose supplementation—BSA by sequestering co‐purified polyphenolic and polysaccharide inhibitors (Kreader 1996), trehalose by thermally stabilising Taq polymerase (Spiess et al. 2004)—suggesting that inhibition was an important contributor to failure in that subset. For minute *Vertigo* specimens, where repeat extraction may be undesirable or impossible, this is practically useful because simple reaction‐level optimisation can sometimes recover otherwise marginal lysates without re‐extraction.

In contrast, GeneJET Genomic underperformed by both purity metrics and amplification success, indicating that this workflow is less suited to minute ethanol‐preserved *Vertigo* material. Its high elution volume (100 μL) may further dilute already‐limited DNA from sub‐milligramme tissue input, compounding the effect of poor inhibitor removal. Its near‐zero A260/230 values are also consistent with incomplete removal of ethanol and guanidinium salts carried over from the wash steps, both of which absorb near 230 nm; an additional dry centrifugation of the column before elution would be a straightforward way to test this, although we did not evaluate a modified protocol here. More broadly, the results reinforce that fluorometric quantification is essential for low‐input molluscan extracts because NanoDrop values can be dominated by non‐DNA absorbance—a point underscored by Winnepenninckx et al. (1993), who noted that CTAB‐polysaccharide complexes can inflate spectrophotometric readings even when effectively removed from the final eluate. Extraction‐method choice should be driven by study aim rather than yield alone. When shell retention is essential, the non‐destructive HotSHOT protocol remains defensible, especially if shorter amplicons or replicate PCRs are acceptable. This reflects a broader trend toward minimally invasive DNA sampling in molluscan conservation biology: from foot‐mucus and periostracum sampling (Armbruster et al. 2005) and FTA‐card swabbing (Leung et al. 2024), through whole‐shell soaking for historical DNA recovery (Ferreira et al. 2020; Martin et al. 2021; Goulding 2021), to shotgun sequencing of subfossil material (Psonis et al. 2022). Our non‐destructive HotSHOT approach occupies a practical niche between these extremes—more invasive than mucus swabbing but less so than shell dissolution—and is specifically tailored to the minute body size that makes surface‐sampling impractical in Vertiginidae. When destructive sampling is possible and broad multilocus success is the priority, DNeasy Blood & Tissue and CTAB + PVP‐40 provide the most dependable options within this dataset.

### 4.1 What Drives the Differences Between Methods

The differences summarised above are not arbitrary, and it is worth stating what we think produces them. DNeasy Blood & Tissue gave the highest fluorometric concentration (median 37.50 ng/μL) despite a 100 μL total elution, which indicates genuinely higher recovery rather than a concentration artefact. QIAamp DNA Micro reached a lower concentration (median 12.50 ng/μL) despite a 20–30 μL elution, pointing to lower absolute recovery from crushed whole specimens: the kit is optimised for cell‐poor liquid inputs rather than for chitinous and calcified homogenates, and the carrier RNA it requires also perturbs absorbance‐based purity estimation, consistent with the highly variable A260/230 values observed here (median 3.69, IQR 1.44–6.25). GeneJET Genomic performed worst on both purity and amplification, which we attribute to a 100 μL elution diluting an already minimal template combined with less effective removal of 230 nm‐absorbing contaminants. CTAB + PVP‐40 achieved the highest A260/280 because organic‐phase partitioning removes protein efficiently, while its lower concentration reflects losses during precipitation from sub‐milligramme input. The two HotSHOT variants differ from each other chiefly in contaminant carry‐over, since the non‐destructive variant never releases crushed shell matrix and periostracal material into the lysate. Because elution volumes differ by a factor of five across methods, concentration alone understates recovery for the low‐elution kits; expressed as total DNA per specimen, the ranking is DNeasy Blood & Tissue ≫ GeneJET Genomic > QIAamp DNA Micro ≈ CTAB + PVP‐40 ≈ destructive HotSHOT ≫ non‐destructive HotSHOT.

### 4.2 Silica‐Column Chemistry: Purity, Recovery and the Limits of the Expected Trade‐Off

Silica‐column purification is conventionally expected to deliver cleaner but less abundant DNA than crude lysis, and our data both support and qualify that expectation. The purity advantage was realised: DNeasy Blood & Tissue and QIAamp DNA Micro were the only methods with A260/280 in the ideal range (median 1.79 for both), and DNeasy also gave an acceptable A260/230 (median 1.83), consistent with chaotropic‐salt binding and ethanol washing removing the proteins and polysaccharides that CTAB and alkaline lysis leave behind. The expected yield penalty, however, did not materialise for DNeasy, which produced both the highest purity and by a wide margin the highest concentration. The reason is that the yield penalty usually attributed to silica columns arises from binding capacity and from irreversible retention of a fraction of the bound DNA, neither of which is limiting at the sub‐nanogram‐to‐nanogram loads typical of Vertiginidae. The penalty is instead visible in GeneJET, where a 100 μL elution over a column optimised for larger loads dilutes the eluate, and in QIAamp DNA Micro, where recovery from a solid crushed homogenate falls short of what the kit achieves on its intended input types. One trade‐off is not negotiable: silica columns remove inhibitors well but cannot preserve the shell, so purity and voucher retention cannot be optimised simultaneously by that route.

### 4.3 The Shell‐Preserving Protocol: Performance Envelope and Practical Use

Because the shell‐preserving protocol is the option this study exists to make available, its working envelope deserves to be stated plainly. It yields the lowest DNA concentration of any method tested (median 0.17 ng/μL) yet amplified 10/10 for the COI mini‐barcode, 9/10 for both ITS loci and 8/10 for full‐length COI. Shorter targets should therefore be preferred where the choice exists, and a failed reaction is better addressed by repeating the PCR with BSA and trehalose than by concluding that the extract is exhausted. All ten shells were recovered intact with apertural dentition legible, so the voucher is genuinely retained rather than merely survived. Two alternatives suggest themselves and neither is applicable at this body size. Removing a fragment of shell rather than recovering the whole shell would not help, because the DNA source here is the soft body inside the shell rather than the shell itself, so a fragment would not release the tissue; and at a shell height of 1.6–2.7 mm with a wall on the order of tens of micrometres thick, controlled removal of a fragment without fracturing the shell or destroying the apertural dentition is not realistic. The surface‐sampling approaches developed for larger gastropods—recovering DNA from the periostracum fraction and from pedal mucus (Armbruster et al. 2005; Efstratiou et al. 2025) – fail for the same reason that FTA‐card swabbing does (Leung et al. 2024): both require a surface large enough to abrade or an animal that can be induced to crawl and secrete measurably, and in Vertiginidae neither is available without the handling damage those methods exist to avoid. Whole‐specimen alkaline lysis with shell recovery occupies precisely the niche below the size threshold at which surface sampling stops working. What should transfer to other taxa is not the protocol as written but the conditions under which it is the right choice: sub‐milligramme tissue mass, a high ratio of mucus to tissue, and a calcified structure that carries diagnostic value and must survive.

The Sanger sequencing verification on three Habitats Directive Annex II species confirms that even the shell‐preserving non‐destructive HotSHOT protocol—which yielded the lowest fluorometric extract concentrations in the main comparison—produces Sanger‐sequenceable DNA sufficient for molecular identification of *V. angustior*, *V. geyeri* and *V. moulinsiana*.

### 4.4 Limitations

Several features of the study design constrain how far these results should be generalised, and the following points should be borne in mind when applying them. Sample sizes were modest (*n* = 10 per method), and several method × locus combinations reached either 0% or 100% amplification success, limiting formal inference. The Bioanalyzer comparison was performed on a separate set of specimens and summarised only within a selected 200–1000 bp window; fragments outside that window, including high‐molecular‐weight DNA, were not incorporated into the comparative metric, and profiling was restricted to three of the six methods. A design normalising template mass rather than template volume across methods would answer the complementary question of how a fixed quantity of DNA performs, and would be a useful follow‐up. The HotSHOT rescue assay was a selected pilot follow‐up on initially problematic extracts and should not be interpreted as an unbiased estimate of method‐wide rescue gains. Shell condition after non‐destructive extraction was assessed qualitatively, and long‐term morphological stability after alkaline heat treatment was not evaluated. We also did not test whether an additional dry centrifugation step would improve GeneJET purity. Specimens spanned a 17‐year collection window (2008–2025); ethanol replacement frequency and storage history were not systematically recorded, so potential confounding by specimen age or preservation history could not be fully disentangled. The ideal design for a comparison of this kind would draw all specimens from a single collecting event with identical post‐sampling treatment; that was not achievable for a taxon restricted to protected fen habitats, and it remains the appropriate design for a follow‐up study.

For destructive extraction of minute *Vertigo* specimens, DNeasy Blood & Tissue gave the best overall balance of DNA concentration, acceptable purity and consistent multilocus amplification, while CTAB + PVP‐40 provided the strongest lower‐cost alternative. QIAamp DNA Micro also performed very well, but at the highest reagent cost. The shell‐preserving non‐destructive HotSHOT protocol produced substantially lower extract concentrations yet still supported high PCR success, making it the preferred option when preservation of the shell voucher is essential—particularly for the legally protected Vertiginidae targeted in Habitats Directive monitoring. Overall, method selection should be guided jointly by voucher‐preservation needs, target amplicon length, cost tolerance and whether simple reaction‐level optimisation is acceptable, rather than by spectrophotometric DNA estimates alone. Given that the most comprehensive molecular study of *Vertigo* to date required destruction of all 424 examined shells (Nekola et al. 2018), the availability of a non‐destructive alternative has immediate relevance for future phylogenetic and conservation‐genetic studies that seek to integrate molecular and morphological data from the same individuals.

## Author Contributions

**Benediktas Jukonis:** conceptualization (lead), data curation (lead), formal analysis (lead), investigation (lead), methodology (lead), visualization (lead), writing – original draft (lead), writing – review and editing (equal). **Jekaterina Havelka:** investigation (lead), writing – review and editing (equal). **Grita Skujienė:** resources (lead), supervision (lead), writing – review and editing (equal).

## Funding

This work was supported by Vilnius University and Directorate‐General XII, Science, Research and Development, LIFE16IPE/LT/016.

## Disclosure

Benefit‐Sharing Statement: The specimens used in this study were collected in Lithuania under national regulations. Lithuania ratified the Nagoya Protocol on 2 October 2020. The research involved native species from habitats within Lithuanian territory; no access to genetic resources from other Nagoya Protocol signatory countries was required. No monetary benefits arose from this research. Non‐monetary benefits include the publication of DNA sequences in GenBank (accessions PZ227160‐PZ227162, PZ235182 and PZ236467), freely accessible to the global research community, and the development of a non‐destructive extraction protocol applicable to conservation‐genetic monitoring of EU Habitats Directive Annex II species.

## Conflicts of Interest

The authors declare no conflicts of interest.

## Supporting information


**Table S1:** Step‐by‐step protocols for the six DNA extraction methods compared in the main study.
**Table S2:** Primer names, sequences, source references and expected amplicon lengths for the four loci amplified in this study (COI barcode, COI mini‐barcode, ITS1, ITS2).
**Table S3:** Allocation of the 60 Vertigo antivertigo specimens used in the six‐method comparison across the four approximately five‐year collection periods.
**Figure S1:** Bidirectional ITS1 sequence quality for the three EU Habitats Directive Annex II Vertigo species extracted with the shell‐preserving non‐destructive HotSHOT protocol.

## Data Availability

The Zenodo record (https://doi.org/10.5281/zenodo.21984159) contains summary tables for QuantiFluor concentrations, NanoDrop purity ratios, PCR amplification outcomes, Bioanalyzer region summaries, cost estimates, specimen‐period allocation and five GenBank accession records. Specimen‐period allocation is also reported in Table S3, and bidirectional read‐quality metrics are reported in Figure S1. The generated sequences have been deposited in GenBank: PZ227160 (*V. angustior* ITS1), PZ227161 (*V. geyeri* ITS1), PZ227162 (*V. moulinsiana* ITS1), PZ236467 (*V. antivertigo* ITS1) and PZ235182 (*V. antivertigo* ITS2).
