# Supplementary material for: DNA Extraction Optimisation for Minute Land Snails of Vertigo Müller, 1773 (Gastropoda: Vertiginidae): A Comparative Evaluation of Six Methods, Including a Non‐Destructive Shell‐Preserving Protocol
Source: Ecol Evol. 2026 Sep 4;16(9):e74295. doi: 10.1002/ece3.74295 (PMC13542995; doi:10.1002/ece3.74295)
Supplement: Supplementary file 1 [file ECE3-16-e74295-s001.zip › Jukonis_etal_Vertigo_Supplementary_FINAL_v2.docx]

**Supporting Information**

*DNA extraction optimisation for minute land snails of Vertigo Müller, 1773 (Gastropoda: Vertiginidae): a comparative evaluation of six methods, including a non-destructive shell-preserving protocol*

Benediktas Jukonis, Jekaterina Havelka, Grita Skujienė

This Supporting Information file accompanies the article and is intended to facilitate reproducibility of the extraction and amplification workflow. It contains: (i) Table S1 – detailed step-by-step extraction protocols for all six methods compared in the main study; (ii) Table S2 – primer names, sequences, source references and expected amplicon lengths for the four loci targeted; (iii) Table S3 – the allocation of specimens across collection periods and extraction methods; and (iv) Figure S1 – bidirectional ITS1 sequence quality for the three EU Habitats Directive Annex II Vertigo species. Archived summary data for the extraction-method comparisons are deposited at Zenodo (https://doi.org/10.5281/zenodo.21984159); specimen-period allocation is included in the workbook and also reported in Table S3, while bidirectional sequence-quality summaries are provided in Figure S1.

**Table S1.** Step-by-step protocols for the six DNA extraction methods compared in the main study. Buffer compositions, incubation conditions, and quality-control steps are itemised to permit direct replication.

| **Parameter / step** | **Specification** |
| --- | --- |
| **Method 1. HotSHOT (destructive)** | |
| Tissue input | Whole individual (soft body plus shell) of one Vertigo antivertigo specimen (< 1 mg); ethanol-preserved (96%) |
| Pre-lysis | Specimens air-dried 5 min after removal from ethanol; shells crushed in 0.2 ml PCR tubes using single-use sterile micropestles (one per specimen); work surface decontaminated with 10% sodium hypochlorite between samples |
| Lysis buffer | 50 µL of 25 mM NaOH |
| Incubation | 95 °C for 30 min in a thermocycler |
| Neutralisation | Cool to 10 °C; add 5 µL of 1 M Tris-HCl (pH 8.0) |
| Centrifugation | 14,000 × g for 10 min at room temperature |
| Storage | Supernatant used directly or stored at −20 °C |
| Hands-on time | ≈ 40 min per batch of up to 10 samples |
| Reference | Adapted from Truett et al. (2000) |
| **Method 2. HotSHOT (non-destructive, shell-preserving)** | |
| Tissue input | Intact individual (snail within shell); no crushing |
| Lysis buffer | 50 µL of 25 mM NaOH |
| Incubation | 95 °C for 30 min in a thermocycler |
| Shell recovery | After lysate cools, retrieve emptied shell under stereomicroscope using fine sterile forceps (Dumont No. 5 or equivalent, ethanol-flame-sterilised between specimens); position forceps beneath the aperture to cradle rather than grip the alkaline-weakened shell. For very fragile shells, aspirate lysate with a micropipette (50 µL) and tip the shell onto a clean glass slide |
| Shell rinse | Twice with 96% ethanol to remove residual NaOH; air-dry at room temperature |
| Voucher archival | Store shell individually in a labelled 1.5 mL tube as a morphological voucher |
| Neutralisation | Add 5 µL of 1 M Tris-HCl (pH 8.0); centrifuge at 14,000 × g for 10 min |
| Notes | Alkaline heat exposure may partially degrade the periostracum and conchiolin matrix; handle recovered shells with particular care. In this study all 10 recovered shells remained structurally intact and apertural dentition was clearly visible (see main manuscript Fig. 6) |
| **Method 3. Modified CTAB + PVP-40** | |
| Extraction buffer | 2% w/v CTAB; 100 mM Tris-HCl pH 8.0; 1.4 M NaCl; 20 mM EDTA; 1% w/v PVP-40 (added to sequester polyphenolic compounds and glycosaminoglycans) |
| Supplementation | 50 µL of 0.1 M DTT per 200 µL buffer (≈ 20 mM final) for complete reduction of disulfide-linked mucus glycoproteins; 5 µL of proteinase K (20 mg/mL) |
| Homogenisation | Manual disruption of the whole individual (soft body plus shell) with a sterile micropestle in 200 µL buffer |
| Lysis | 60 °C for 2 h with continuous agitation at 800 rpm in a thermomixer |
| Organic extraction | Add equal volume (~250 µL) phenol:chloroform:isoamyl alcohol (25:24:1); invert gently 5–10 min (no vortexing, to avoid shearing); centrifuge 12,000 × g for 15 min at room temperature; recover upper aqueous phase |
| Precipitation | Add 0.1 vol 3 M sodium acetate (pH 5.2); 0.6 vol cold isopropanol; 1 µL glycogen (20 mg/mL) carrier. Incubate at −20 °C ≥ 1 h; centrifuge 12,000 × g for 30 min at 4 °C |
| Wash | 500 µL cold 75% ethanol; air-dry pellet 5 min until translucent |
| Resuspension | 15–20 µL TE buffer overnight at 4 °C |
| Reference | Modified from Chakraborty et al. (2020); PVP-40 inclusion per Porebski et al. (1997) |
| **Method 4. GeneJET Genomic DNA Purification Kit (Thermo Fisher Scientific)** | |
| Lysis | Whole individual (soft body plus shell) crushed before overnight proteinase K digestion at 56 °C; manufacturer's tissue protocol |
| Column purification | Per manufacturer's instructions |
| Elution | 100 µL elution buffer |
| **Method 5. DNeasy Blood & Tissue Kit (Qiagen)** | |
| Lysis | Whole individual (soft body plus shell) crushed before overnight lysis in Buffer ATL with proteinase K at 56 °C; manufacturer's tissue protocol |
| Column purification | Per manufacturer's instructions |
| Elution | Dual elution: 2 × 50 µL Buffer AE pre-warmed to 70 °C |
| **Method 6. QIAamp DNA Micro Kit (Qiagen)** | |
| Lysis | Whole individual (soft body plus shell) crushed before overnight digestion at 56 °C; carrier RNA added per manufacturer's recommendation to maximise recovery from minimal input |
| Column purification | Per manufacturer's instructions; low-volume design optimised for sub-microgram inputs |
| Elution | 20-30 µL Buffer AE pre-warmed to 70 °C |
| **Common quality-control steps (all methods)** | |
| Negative controls | Reagent-only extraction blank in every batch; no-template (water) PCR control in every amplification run |
| DNA quantification | QuantiFluor® dsDNA System (Promega) on a fluorometer per manufacturer's instructions; NanoDrop 2000 (Thermo Fisher Scientific) for A260/280 and A260/230 ratios |
| Fragment-size profiling | Agilent 2100 Bioanalyzer, High Sensitivity DNA Assay; region analysis 200–1,000 bp |
| Storage | Eluates stored at −20 °C; long-term archival at −80 °C |

**Table S2. Primer names, sequences, source references and expected amplicon lengths for the four loci amplified in this study (COI barcode, COI mini-barcode, ITS1, ITS2). Thermal cycling conditions and reaction setup are given in full in the Materials and Methods of the main article and are not duplicated here.**

| **Locus** | **Primer** | **Sequence (5′→3′)** | **Expected length** | **Reference** |
| --- | --- | --- | --- | --- |
| COI barcode | LCO1490 (F) | GGTCAACAAATCATAAAGATATTGG | ≈ 658 bp | Folmer et al. (1994) |
| COI barcode | HCO2198 (R) | TAAACTTCAGGGTGACCAAAAAATCA | ≈ 658 bp | Folmer et al. (1994) |
| COI mini-barcode | mlCOIintF (F) | GGWACWGGWTGAACWGTWTAYCCYCC | ≈ 313 bp | Leray et al. (2013) |
| COI mini-barcode | jgHCO2198 (R) | TAIACYTCIGGRTGICCRAARAAYCA | ≈ 313 bp | Geller et al. (2013) |
| ITS1 | ITS1 (F) | TCCGTAGGTGAACCTGCGG | ≈ 600 bp | White et al. (1990) |
| ITS1 | ITS2 (R) | GCTGCGTTCTTCATCGATGC | ≈ 600 bp | White et al. (1990) |
| ITS2 | ITS3 (F) | GCATCGATGAAGAACGCAGC | ≈ 450–600 bp | White et al. (1990) |
| ITS2 | ITS4 (R) | TCCTCCGCTTATTGATATGC | ≈ 450–600 bp | White et al. (1990) |

**Notes.** F = forward primer; R = reverse primer. Degenerate base codes: W = A/T; Y = C/T; R = A/G; I = inosine. PCRs were carried out with Platinum II Hot-Start PCR Master Mix (2×; Thermo Fisher Scientific). No-template (water) negative controls were included in every amplification run. Amplification success was scored by the presence of a band of expected size on 1.5% agarose / 1× TAE gels stained with ethidium bromide; fragment sizes were estimated using FastRuler Low Range DNA Ladder (Thermo Fisher Scientific, cat. SM1103).

**Table S3. Allocation of the 60 Vertigo antivertigo specimens used in the six-method comparison across the four approximately five-year collection periods. Specimens were grouped by collection period on the basis of label data and allocated by block randomisation stratified by period; every method received an identical 3 : 2 : 2 : 3 distribution from oldest to most recent material (n = 10 per method).**

| **Extraction method** | **2008–2012** **(oldest)** | **2013–2017** | **2018–2022** | **2023–2025** **(most recent)** | **Total** |
| --- | --- | --- | --- | --- | --- |
| HotSHOT (destructive) | 3 | 2 | 2 | 3 | 10 |
| HotSHOT (non-destructive) | 3 | 2 | 2 | 3 | 10 |
| CTAB + PVP-40 | 3 | 2 | 2 | 3 | 10 |
| GeneJET Genomic | 3 | 2 | 2 | 3 | 10 |
| DNeasy Blood & Tissue | 3 | 2 | 2 | 3 | 10 |
| QIAamp DNA Micro | 3 | 2 | 2 | 3 | 10 |
| **Total** | **18** | **12** | **12** | **18** | **60** |

**Figure S1. Bidirectional ITS1 sequence quality for the three EU Habitats Directive Annex II Vertigo species extracted with the shell-preserving non-destructive HotSHOT protocol. (a–c) Reverse-read Sanger chromatograms (positions 80–144 bp of the quality-trimmed read) for (a) Vertigo angustior (BJ-2), (b) Vertigo geyeri (BJ-3) and (c) Vertigo moulinsiana (BJ-4); four-channel trace (A = green, C = blue, G = black, T = red), base calls, and a per-base Phred quality strip (red–yellow–green, Q = 0–60). (d) Full-read Phred quality profiles (10 bp sliding-window mean) for forward (dashed) and reverse (solid) reads. (e) Ratio of secondary to primary chromatogram peak height at every base called at Q ≥ 20; the dashed line marks the 30% threshold conventionally used to score a double peak. (f) Summary of read length, quality, ambiguous base calls, positions exceeding the 30% secondary-peak threshold, forward–reverse identity over the aligned overlap, and the number of positions at which an above-threshold secondary peak is present in both reading directions – the signature expected of a genuine heterozygous site.**
